# Efficacy of Therapeutic Endoscopy for Gastrointestinal Lesion (GI): A network meta-analysis

**DOI:** 10.12669/pjms.35.2.636

**Published:** 2019

**Authors:** Tian-xi Wang, Jun Zhang, Li-hong Cui, Jing-jing Tian, Rongna Wei

**Affiliations:** 1 *Tian-xi Wang, Department of Gastroenterology, Tianjin Nankai Hospital, Tianjin, 300100, China*; 2 *Jun Zhang, General Medicine, Tianjin Beichen Hospital, Tianjin, 300401, China*; 3 *Li-hong Cui, Department of Gastroenterology, Tianjin Nankai Hospital, Tianjin, 300100, China*; 4 *Jing-jing Tian, Department of Gastroenterology, Tianjin Nankai Hospital, Tianjin, 300100, China*; 5 *Rongna Wei, Department of Gastroenterology, Tianjin Nankai Hospital, Tianjin, 300100, China*

**Keywords:** Endoscopic therapy, Gastrointestinal Lesion, Bleeding

## Abstract

**Objective:**

Endoscopic therapy can reduce the risks of rebleeding, continued bleeding, need for surgery, and mortality. The objective of this systematic review was to compare the different modalities of endoscopic therapy for GI bleeding.

**Methods:**

Studies were identified by searching electronic databases MEDLINE. We selected all available clinical studies published after 2000 that assessed efficacy and/or safety of different endoscopic hemostatic techniques in treating GI bleeding. The outcomes evaluated included initial hemostasis, rebleeding rate, and 30-day all-cause mortality. Network meta-analyses were performed to summarize the treatment effects.

**Results:**

Total 20 studies involving 1845 patients were evaluated. Ten different treatment categories including mechanic, ablative, injection, and combined therapy were compared in our analysis in terms of their efficacy in stopping bleeding and complications. Band ligation [rate: 0.757; 95% Credible Interval (0.565, 0.887)] and injection therapy [rate: 0.891; 95% CI (0.791, 0.944)] had inferior efficacy in attaining initial hemostasis compared to others. Combined therapy of band ligation and HPC and hemoclip may represent the best options for preventing rebleeding and mortality respectively. No significant difference was found among other treatments in terms of complications.

**Conclusions:**

We recommend the application of hemoclips in treating GI bleeding due to its high hemostasis efficacy and low risk of 30-day mortality.

## INTRODUCTION

Gastrointestinal lesions are defined as abnormal vascular dilations that communicate capillaries and veins in the walls of the digestive tract, whose clinical presentation varies from chronic occult bleeding to severe gastrointestinal bleeding.^1^ They include arterio-venous malformations as angiodysplasia and Dieulafoy’s lesion, venous ectasias (multiple phlebectasias and haemorroids), teleangiectasias which can be associated with hereditary hemorrhagic teleangiectasia (HHT), Turner’s syndrome and systemic sclerosis, haemangioma’s, angiosarcoma’s and disorders of connective tissue affecting blood vessels as pseudoxanthoma elasticum and Ehlers-Danlos’s disease.^2^ Preventing GI bleeding through early diagnosis or effectively reducing the rate of GI bleeding through medical therapy becomes crucial in clinical settings.

Therapeutic endoscopy is the primary diagnostic and therapeutic treatment modality for acute GI bleeding. It can be carried out through argon plasma coagulation, electrocoagulation, photocoagulation, endoscopic clips, or injection sclerotherapy. The efficacy of therapeutic endoscopy depends on findings of stigmata of recent hemorrhage (SRH).^3^ Commonly-seen endoscopic therapies include injection, ablation, and mechanical therapy. Studies showed that monotherapy reduces the risk of rebleeding in patients with peptic ulcer disease with major SRH to about 20%. Combination therapy, especially injection followed by either ablation or mechanical therapy, is generally recommended to further reduce the risk of rebleeding to about 10%.^4^–^6^ and has been associated with increasing nonsteroidal antiinflammatory drug use and the high prevalence of Helicobacter pylori infection in patients with peptic ulcer bleeding. Rapid assessment and resuscitation should precede the diagnostic evaluation in unstable patients with severe bleeding. Risk stratification is based on clinical assessment and endoscopic findings. Early upper endoscopy (within 24 hours of presentation. In this study, we performed a meta-analysis and sought to determine: 1). How the efficacy of therapeutic endoscopy changes over different endoscopic therapies. 2). What are the potential benefits of endoscopic therapies in reducing re-bleeding, adverse events and others.

## METHODS

### Literature Search

We conducted a systematic literature search of MEDLINE by combining the keywords “Gastrointestinal”, “lesions”, “bleeding”, “Therapeutic endoscopy” or “endoscopic therapy”, “Injection”, “Ablative”, “Mechanic”, “efficacy” with a time period between 2005 to 2018. Unpublished preliminary results were also checked by search on ClinicalTrials.gov. Articles were limited to those published in English. In addition, the references of the retrieved articles were also carefully reviewed by two researchers to identify potentially relevant and eligible studies. Abstracts of citations identified from the literature search were reviewed by three of the reviewers who then independently extracted all relevant data. Any disagreements were resolved by consensus agreement.

### Inclusion and Exclusion Criteria

The inclusion criteria were: (1) English literature; (2) the study design aimed at evaluating efficacy of endoscopic treatment, such as hemoclips, injection therapy and thermocoagulation, in preventing GI bleeding and reducing adverse events; (3) the article has available data for extraction and reported at least one clinical outcome or perioperative data; (4) acute bleeding from peptic ulcer or Dieulafoy lesions was made endoscopically; (5) the sample size should be above ten patients for both groups; and (6) the full text was available; (7) at least one of the following outcomes was reported: initial hemostasis after first endoscopic therapy; rebleeding; definitive hemostasis, surgical intervention; mortality; Duplicated studies, non-human studies, case reports, and review articles were excluded.

### Data Extraction

Study characteristics including year of publication, the first author, country, study design, sample size, type of endoscopic treatment, mean age, concomitant diseases, and location of GI bleeding were extracted from eligible studies. Primary outcome of this study is definitive hemostasis defined as successful control of bleeding after the the endoscopic therapy until the end of follow up. Other outcomes include rebleeding (clinical evidence of recurrent bleeding after the treatment), surgery and death from any cause (30-day mortality or “in-hospital” mortality).

### Statistical Analyses

All statistical analyses were performed by R version 3.4.1 using pcnetmeta3. The binary outcome was summarized using absolute risk or odds ratio (OR) and 95% credible interval. Continuous outcomes were summarized by the posterior mean difference and 95% CIs. For indirect pairwise meta-analyses, the network meta-analysis was carried out to investigate the robustness of our findings and to combine both direct and indirect evidence about any procedure among endoscopic therapies. Hierarchical Bayesian models using Markov Chain Monte Carlo (MCMC) methods were implemented in NMA. The significance of the difference of direct or indirect comparison was visualized by contrast plots. The surface under the cumulative ranking curves (SUCRA), which is a numeric presentation of the overall ranking and presents a single number associated with each treatment, were created based on ranked probability. SUCRA values range from 0 to 100%. The higher the SUCRA value, and the closer to 100%, the higher the likelihood that a therapy is in the top rank or one of the top ranks; the closer the SUCRA value is to 0, the more likely that a therapy is in the bottom rank, or one of the bottom ranks.

## RESULTS

### Study Selection

**Fig.1:**
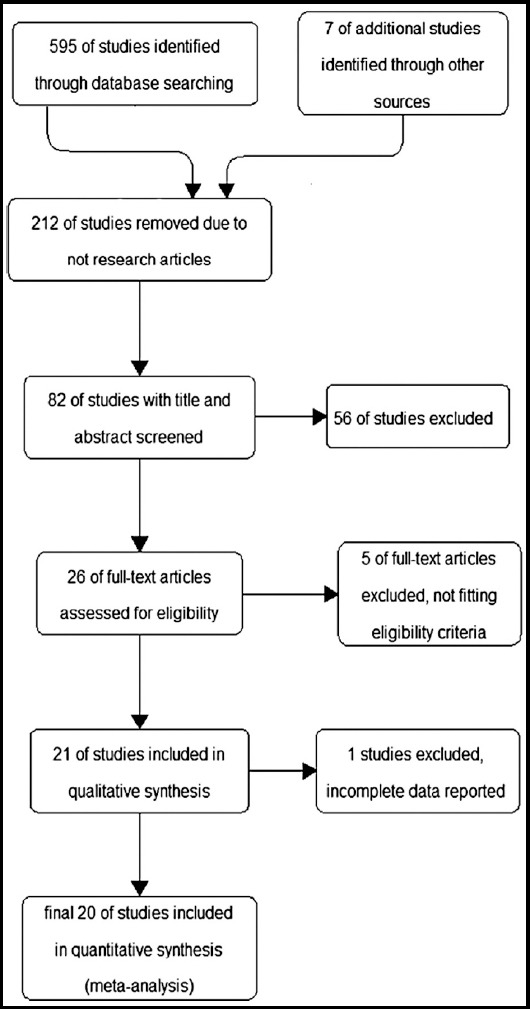
Consort Diagram to Show Study Selection Process.

Searches of MEDLINE yielded 595 records, and manual searches of bibliographies of reviews, meta-analyses, and other trial publications identified an additional seven articles (Fig.1). After removal of duplicates and non-research studies, 82 titles and abstracts were screened for eligibility, and 26 article texts were reviewed for inspecting the integrity and quality of data. 20 studies^7^–^26^ but their efficacy can be suboptimal in patients with complex bleeding lesions. The over-the-scope clip (OTSC were included in our network meta-analysis.

### Study Characteristics

The characteristics including author, publication year, country or region for conducting studies, total sample size, mean age, percentage of male sex, treatments, and study endpoints (study outcomes) of the 20 eligible studies are summarized in Table-I. As shown in Table 1, 11 were randomized clinical trials (RCTs), two prospective observational studies, and seven retrospective analyses. The total sample size ranged from 10 to 198. The mean age ranged from 52 to 72 years old. The male predominance was appeared in all included studies (Male sex > 50%). Most of the studies had a follow-up of one month. The study treatments comprised injection therapy, over-the-scope clip (OTSC), hemoclip, argon plasma coagulation (APC), heat probe coagulation (HPC), band ligation, and combined therapy of any two of the above treatments. The primary and secondary outcomes included initial hemostasis, rebleeding, 30-day all-cause mortality, and other complications.

**Table-I:** Study Characteristics.

ID	Country/ Region	Method	Study	Sample Size	Mean Age	Male Sex (%)	Follow-up	Measures	Category
Manta et al, 2013	Italy	OTSC	Observational	30	64	47	1 month	Hemostasis and complications	Mechanic
Manno et al, 2016	Italy	OTSC	Observational	40	69	82.5	1 month	Hemostasis, complications, and mortality	Mechanic
Wedi et al, 2017	Germany	OTSC	Retrospective	100	72	72	6 months	Hemostasis, complications, and mortality	Mechanic
Wander et al, 2018	US	Hemoclip	Retrospective	178	/	65.2	1 month	Hemostasis and complications	Mechanic
Akin et al, 2017	Turkey	HPC and APC with epinephrine injection	Retrospective	97	59.5	73.2	1 month	Hemostasis, complications, and mortality	Ablative + Injection
Hosoe et al, 2009	Japan	Hemoclip	Retrospective	198	63.6	77.8	/	Hemostasis and complications	Mechanic
Wang et al, 2015	Taiwan	APC with Injection vs Injection	RCT	116	63.7	70	1 month	Hemostasis, complications, and mortality	Injection vs Combined
Wang et al, 2009	Taiwan	APC vs Injection	RCT	135	68	71.3	1 month	Hemostasis, complications, and mortality	Ablative vs Injection
Li et al, 2014	Taiwan	APC with Injection vs Injection	Retrospective	120	66.85	47.5	1 month	Hemostasis, complications, and mortality	Ablative vs Combined
Chau et al, 2003	China	HPC+Injection vs APC+Injection	RCT	192	62.7	67.6	1 month	Hemostasis, complications, and mortality	Combined
Thosani et al, 2014	US	APC+Injection vs Injection	Retrospective	10	58	80	1 month	Hemostasis, complications, and mortality	Ablative vs Combined
Brandler et al, 2018	US	OTSC	Retrospective	67	70.9	56.7	1 month	complications	Mechanic
Lo et al, 2001	China	Injection vs Band Ligation	RCT	60	56.55	76.7	1 year	Hemostasis, complications, and mortality	Injection vs Mechanic
Tantau et al, 2014	Romania	Injection vs Band Ligation	RCT	37	60.22	56.8	2 years	Hemostasis, complications, and mortality	Injection vs Mechanic
Monici et al, 2010	Brazil	Injection + Band Ligation vs Band Ligation + Ablative	RCT	70	72.8	48	2 years	Hemostasis, complications, and mortality	Combined
Luz et al, 2010	Brazil	Injection vs Band Ligation	RCT	100	52	72	6 weeks	Hemostasis, complications, and mortality	Injection vs Mechanic
Ferrari et al, 2005	Brazil	Injection vs Band Ligation	RCT	46	49	78.3	1 year	Hemostasis, complications, and mortality	Injection vs Mechanic
Tan et al, 2006	Taiwan	Injection vs Band Ligation	RCT	97	61	71.3	6 years	Hemostasis, complications, and mortality	Injection vs Mechanic
Lo et al, 2006	Taiwan	Injection vs Injection+Hemoclip	RCT	105	63.5	77	1 year	Hemostasis, complications, and mortality	Injection vs Combined
Saltzman et al, 2005	US	Hemoclip vs Hemoclip+Injection	RCT	47	65.1	65.95	1 month	Hemostasis, complications, and mortality	Hemoclip vs Combined

### Initial Hemostasis

**Fig.2:**
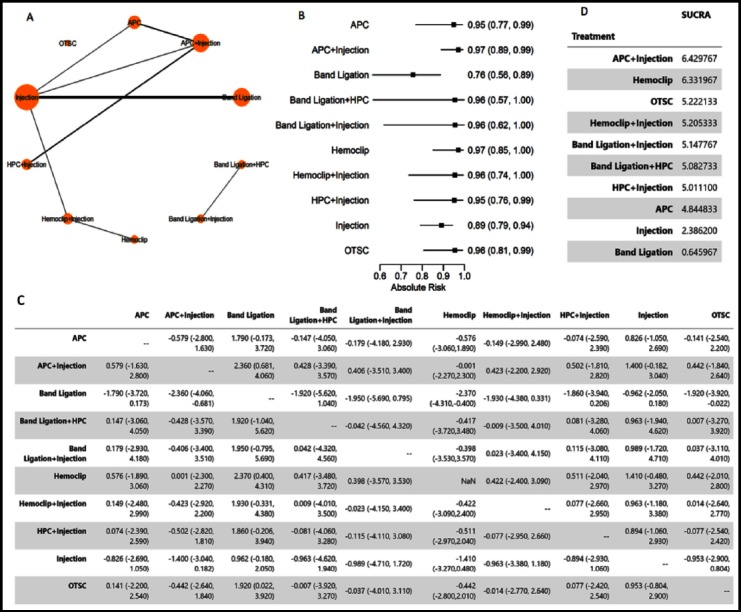
Network Meta-analysis of Endoscopic Therapy for Achieving Initial Hemostasis (A). Network Graph. (B). Plot of Absolute Risk (The higher the better). (C). Head-to-Head Comparisons. (D). SUCRA summary.

The main purpose of this section is to analyze which treatment had superior efficacy in achieving initial hemostasis. A network meta-analysis was conducted. The network graph, plot of head-to-head comparison, and absolute risk plot are shown in Fig.2. As shown by the results, total 10 treatment categories (represented as node) were compared. Each edge between two nodes stands for a direct comparison between the corresponding two treatments (Fig.2A). Band ligation had the worst efficacy in achieving initial hemostasis [Risk ratio: 0.76 (0.56-0.79)]. Head-to-head comparison of log Odds Ratio (OR) with median credible interval (CI) suggested that band ligation had significant inferior hemostatic efficacy compared to combined therapy of APC and injection [2.360 (0.681-4.060)], hemoclip [2.370 (0.400-4.310)], and OTSC [1.920 (0.022-3.920)]. The combined therapy of APC and injection and Hemoclip may represent the best treating modality in controlling bleeding according to SUCRA ranking (Fig.2D).

### Rebleeding

**Fig.3:**
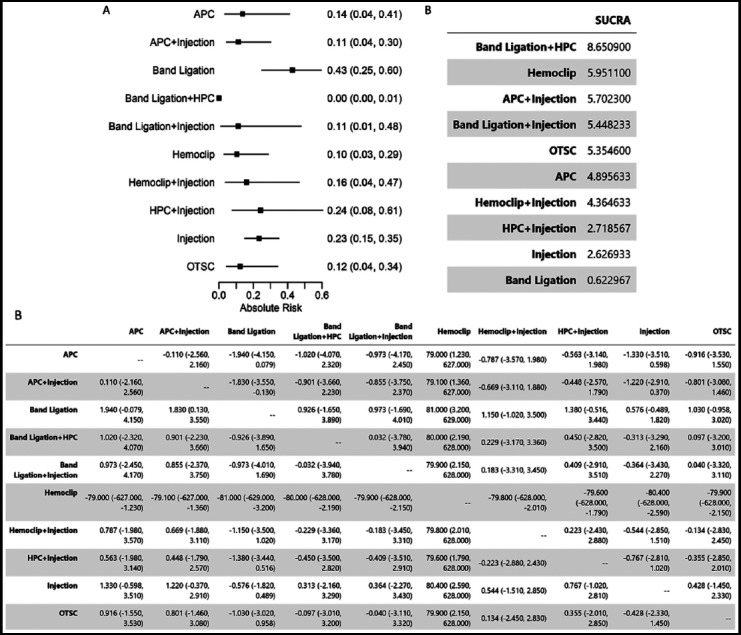
Network Meta-analysis of Endoscopic Therapy for Risk of Rebleeding (A). Plot of Absolute Risk (The lower the better). (B). Head-to-Head Comparisons. (C). SUCRA summary.

Total 19 studies were included in the network meta-analysis for the event of rebleeding. As indicated in Fig.3, the combined therapy of band ligation and HPC had superiority in terms of rebleeding compared to others (Fig.3). Band ligation is associated with the highest risk of rebleeding among all treatments. Interestingly, we found the combined therapy (band ligation + HPC) had the highest value of SUCRA, which indicates that it could be recommend as the best option for having the low rebleeding rate.

### 30-day Mortality

**Fig.4:**
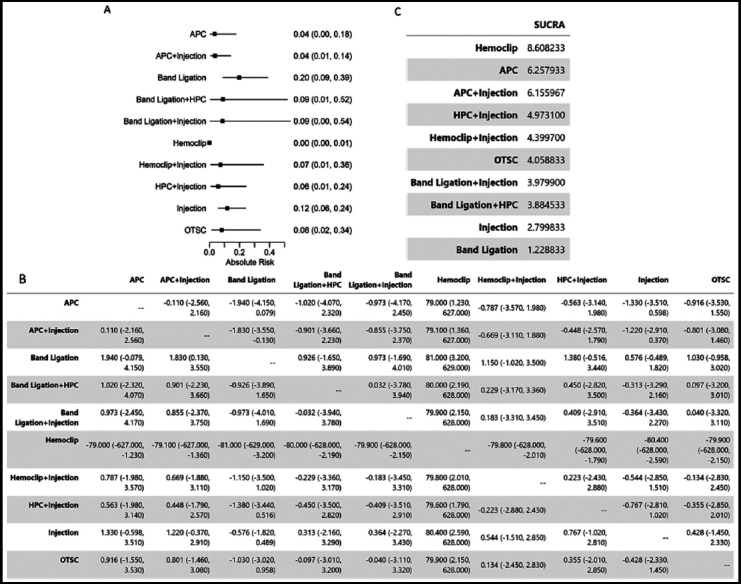
Network Meta-analysis of Endoscopic Therapy for Risk of 30-day Mortality (A). Plot of Absolute Risk (The lower the better). (B). Head-to-Head Comparisons. (C). SUCRA summary.

The results of network meta-analysis of comparing the risk of mortality of different endoscopic therapies is shown in Fig-4. It’s noted that due to a large missing of reporting treatment-related mortality, here we only considered 30-day mortality of all causes. Based on the results, we found there is no significant difference in inducing mortality within 30 days among the treatment modalities other than hemoclip. Hemoclip may represent the best option for GI bleeding treatment in terms of reducing 30-day mortality (Fig.4C).

## DISCUSSION

The most commonly used endoscopic therapies for GI bleeding could be classified into three categories: injection therapy, mechanic therapy, and ablative therapy.^3^ All the above therapies are aimed at preventing continued bleeding or rebleeding while each of them has its own advantage. For instance, injection therapy is easier for administrating and confers a role of serving as an initial agent in controlling GI bleeding. Ablative therapies, either through contact (e.g. HPC) or non-contact method (e.g APC), could achieve hemostasis very quickly by delivering intense energy to coagulate tissue protein in the bleeding site. Mechanic therapy such as hemoclip has advantages in treating patients with coagulopathy by occluding bleeding vessel. In this study, we conducted a network meta-analysis to compare hemostatic efficacy and complications of the different endoscopic therapies. To the best of our knowledge, there is no systematic review which has used network meta-analysis to perform indirect and direct comparison.

In our study, we included 20 studies, which included 1845 patients with diverse country of origin. Most of the studies were RCTs. Except band ligation (0.76) and injection therapy (0.89), nearly all treatments could maintain above 95% rate of initial hemostasis. APC+Injection and hemoclip were recommended as the best options for achieving initial hemostasis based on ranking of SUCRA values. Band ligation+HPC is associated with the lowest risk of re-bleeding while band ligation alone had the highest risk of re-bleeding which suggested that combined therapy had add-on value to band ligation alone. We also considered 30-day mortality rate for each treatment. We found Hemoclip is associated with the lowest risk of mortality while others had no significant difference.

### Limitations of the study

(1) Even though we had included a large amount of RCTs, which were regarded had good study quality, the included retrospective and observational studies may impact the overall study quality and increase study heterogeneity. (2) In our network meta-analysis, we had several treating categories but had one study, which may induce inaccuracy of the results. (3) During our study selection, we included patients with GI bleeding irrespective of the causes and other comorbidities. The severity and etiology of the different studies were ignored. (4) The studies were from very diverse countries or regions. The diverse origins of the patients also may induce a significant heterogeneity.

### Authors’ Contributions

**JZ** designed the study and prepared the manuscript.

**TXW and LHC** Literature search and retrieved data.

**JJT and RNW** collected the data. All authors have read and approved the final manuscript.
